# PERK/NRF2 and autophagy form a resistance mechanism against G9a inhibition in leukemia stem cells

**DOI:** 10.1186/s13046-020-01565-3

**Published:** 2020-04-15

**Authors:** Ji Eun Jang, Ju-In Eom, Hoi-Kyung Jeung, Haerim Chung, Yu Ri Kim, Jin Seok Kim, June-Won Cheong, Yoo Hong Min

**Affiliations:** 1 Department of Internal Medicine, Yonsei University College of Medicine, Seoul, Korea; 2 Avison Biomedical Research Center, Yonsei University College of Medicine, Seoul, Korea

**Keywords:** Leukemia stem cells, G9a, PERK/NRF2, Autophagy, Resistance

## Abstract

**Background:**

The histone methyltransferase G9a has recently been identified as a potential target for epigenetic therapy of acute myeloid leukemia (AML). However, the effect of G9a inhibition on leukemia stem cells (LSCs), which are responsible for AML drug resistance and recurrence, is unclear. In this study, we investigated the underlying mechanisms of the LSC resistance to G9a inhibition.

**Methods:**

We evaluated the effects of G9a inhibition on the unfolded protein response and autophagy in AML and LSC-like cell lines and in primary CD34^+^CD38^−^ leukemic blasts from patients with AML and investigated the underlying mechanisms. The effects of treatment on cells were evaluated by flow cytometry, western blotting, confocal microscopy, reactive oxygen species (ROS) production assay.

**Results:**

The G9a inhibitor BIX-01294 effectively induced apoptosis in AML cell lines; however, the effect was limited in KG1 LSC-like cells. BIX-01294 treatment or siRNA-mediated G9a knockdown led to the activation of the PERK/NRF2 pathway and HO-1 upregulation in KG1 cells. Phosphorylation of p38 and intracellular generation of reactive oxygen species (ROS) were suppressed. Pharmacological or siRNA-mediated inhibition of the PERK/NRF2 pathway synergistically enhanced BIX-01294-induced apoptosis, with suppressed HO-1 expression, increased p38 phosphorylation, and elevated ROS generation, indicating that activated PERK/NRF2 signaling suppressed ROS-induced apoptosis in KG1 cells. By contrast, cotreatment of normal hematopoietic stem cells with BIX-01294 and a PERK inhibitor had no significant proapoptotic effect. Additionally, G9a inhibition induced autophagy flux in KG1 cells, while autophagy inhibitors significantly increased the BIX-01294-induced apoptosis. This prosurvival autophagy was not abrogated by PERK/NRF2 inhibition.

**Conclusions:**

PERK/NRF2 signaling plays a key role in protecting LSCs against ROS-induced apoptosis, thus conferring resistance to G9a inhibitors. Treatment with PERK/NRF2 or autophagy inhibitors could overcome resistance to G9a inhibition and eliminate LSCs, suggesting the potential clinical utility of these unique targeted therapies against AML.

## Background

Most patients with acute myeloid leukemia (AML) achieve complete remission with induction chemotherapy; however, the majority of patients relapse owing to the development of drug resistance [1]. Leukemia stem cells (LSCs) play important roles in the relapse and drug resistance, which hamper complete cure of the disease [2]. Therefore, new therapeutic strategies for effective targeting of LSCs are required to cure AML.

In addition to the recurring cytogenetic and molecular abnormalities, which have been characterized in AML, recent discoveries have highlighted the major role of dysregulated epigenetic mechanisms in AML pathogenesis [3, 4]. Studies have indicated that mutations related to DNA methylation could mediate abnormal self-renewal and differentiation of LSCs, suggesting that drugs targeting epigenetic enzymes may provide a new AML treatment option [5–7]. In contrast to genetic changes, epigenetic modifications are generally reversible, thus providing opportunities for targeted treatment with specific inhibitors [8]. The histone methyltransferase (HMTase) G9a, an epigenetic enzyme w ubiquitously expressed in somatic cells, contributes to the development and progression of various cancers [9–13]. G9a regulates the transcription of multiple genes by primarily catalyzing the dimethylation of histone H3 lysine 9 (H3K9me2) [13] and induces changes in cellular redox homeostasis, leading to a decrease in reactive oxygen species (ROS) production [14]. The G9a protein is accumulated under hypoxic conditions, without an alteration in the levels of G9a transcripts, leading to the epigenetic silencing of specific genes in cancer cells [15, 16]. High levels of G9 expression are associated with unfavorable clinical outcomes, including disease progression, metastasis, development of stem cell-like characteristics, resistance to treatment, and poor survival [17, 18]. Inhibition of G9a suppresses cell proliferation and modulates redox homeostasis, with an increase in ROS generation [14]. It has been shown that G9a inhibition induces cancer cell death via intracellular ROS production [19]. Thus, targeting of G9a has attracted attention as a novel strategy for the treatment of various types of tumors, including leukemia, which are characterized by hypoxic regions [20]. Several recent reports have suggested that this HMTase may be a potential target for epigenetic therapy of AML. Pharmacological and genetic targeting of G9a has been shown to be effective in slowing down the AML cell proliferation and reducing the LSC frequency in a mouse model and human AML cell lines due to the attenuation of HoxA9-dependent transcription [21–23]. San José-Enériz et al. have reported that inhibitors of G9a/DNMTs significantly prolong the survival of AML xenogeneic models [24]. However, the effect of G9a inhibition on LSCs remains elusive. The tolerance of LSCs to epigenetic therapies causes treatment failure, thus hampering the clinical applicability of these therapies, while inhibition of LSC tolerance is considered a promising therapeutic strategy.

The endoplasmic reticulum (ER) has a very specific signaling network system, termed the unfolded protein response (UPR), which allows cells to adapt to stress or activate apoptotic signaling when intracellular protective mechanisms are insufficient [25]. G9a inhibition stimulates caspase-dependent apoptosis and ER stress in human bladder cancer cells [26]. It has been recently shown that the PERK pathway, which is one of the main UPR signaling cascades, acts as a prosurvival ER stress signal in cancer cells and contributes to the development of drug resistance [27]. ROS are sensed by stress sensors in the ER lumen and amplify UPR signaling [28]. PERK inhibits ROS accumulation by phosphorylating and stabilizing NRF2, which regulates the expression of ROS-induced phase II detoxification enzymes, such as glutathione *S*-transferase and HO-1 [29, 30]. The PERK pathway is a reasonable resistance mechanism candidate, given that G9a targeting induces cancer cell death through intracellular ROS production. Autophagy is another cell survival mechanism, which regulates cancer stem cell properties by contributing to the maintenance of stemness, induction of recurrence, and development of resistance to anticancer agents [31].

In this study, we investigated, for the first time, the effect of G9a inhibition on the PERK pathway and autophagy, contributing to LSC survival, and the potential of therapeutic targeting of epigenetic mechanisms. We aimed to elucidate the underlying mechanism of LSC resistance to G9a inhibition and to propose a new therapeutic strategy for the elimination of AML LSCs.

## Materials and methods

### Patients and isolation of AML cells

Human leukemia cells were obtained from diagnostic bone marrow aspirates of patients with de novo AML, who were diagnosed at the Yonsei University Severance Hospital (Seoul, South Korea). This prospective cohort has been registered at ClinicalTrials.gov (NCT02344966). As AML is a heterogeneous disease, patients were stratified into risk groups based on cytogenetic and molecular abnormalities [32]. To minimize the effect of a heterogeneous response to treatment, we used samples from patients with cytogenetically normal AML, without recurrent mutations. After thawing cryopreserved primary AML cells, CD34^+^ leukemia cells were enumerated by flow cytometry (LSR Fortessa; BD Biosciences) using an APC-labeled antibody against the myeloid stem cell marker CD34 (BD Biosciences). Samples from 16 patients with abundant CD34^+^ leukemia cells (> 57% of mononuclear cells) were selected for the experiments (Additional file 1: Table S1). Human adult CD34^+^ hematopoietic stem cells (HSCs) were obtained from healthy sibling donors who donated HSCs for allogeneic stem cell transplantation.

### Cell culture and chemicals

The AML cell lines U937, MOLM-13, and MV4–11 and the LSC-like cell lines KG1, KG1a, and Kasumi-1, which are enriched in cells expressing an LSC phenotype (CD34^+^CD38^−^), were obtained from the American Type Culture Collection. U937, MOLM-13, MV4–11, KG1, and Kasumi-1 cells were cultured in RPMI-1640 medium (Gibco Life Technologies), and KG1a cells were cultured in Iscove’s modified Dulbecco’s medium (Gibco Life Technologies) supplemented with 10% (v/v) fetal bovine serum and penicillin (100 U/mL)/streptomycin (0.1 mg/mL). The G9a inhibitor BIX-01294, the autophagy inhibitors 3-methyladenine (3-MA) and bafilomycin A1, and the NRF2 inhibitor brusatol were purchased from Sigma–Aldrich. The PERK inhibitor GSK2606414 was purchased from Selleckchem. Logarithmically growing cells (2 × 10^5^ cells/mL) were exposed to different concentrations of BIX-01294 alone or in combination with the other drugs. The pan-caspase inhibitor Z-VAD-FMK (R&D Systems) was added to cells prior to the drug addition in some experiments.

### Antibodies

Rabbit polyclonal antibodies against microtubule-associated protein 1 light chain 3 (LC3) (NB100–2220) and p62 (NBP1–48320) were obtained from Novus Biologicals. A rabbit polyclonal antibody against poly (ADP-ribose) polymerase (PARP; 9542), horseradish peroxidase (HRP)-conjugated goat anti-rabbit IgG (7074), and HRP-conjugated goat anti-mouse IgG (7072) were purchased from Cell Signaling Technology. A mouse anti-α-tubulin monoclonal antibody (05–829) was obtained from Merck Millipore. Antibodies against G9a (B9311; Sigma–Aldrich), phospho (p)-PERK (3179; Cell Signaling Technology), PERK (5638; Cell Signaling Technology), p-eIF2α (9721; Cell Signaling Technology), ATF4 (11,815; Cell Signaling Technology), CHOP (SC7351; Santa Cruz Biotechnology), H3K9me1 (AB8896; Abcam), H3K9me2 (AB1220; Abcam), H3K27me1 (AB194688; Abcam), H3K27me2 (9728; Cell Signaling Technology), p-p38 (9211; Cell Signaling Technology), caspase-3 (9662; Cell Signaling Technology), caspase-9 (9502; Cell Signaling Technology), NRF2 (SC722; Santa Cruz Biotechnology), and HO-1 (SC136960; Santa Cruz Biotechnology) were also used.

### Western blotting

Total cell lysates were prepared and analyzed by western blotting, as described previously [33]. Protein samples were recovered in sodium dodecyl sulfate (SDS) buffer and separated by SDS-polyacrylamide gel electrophoresis. The separated proteins were transferred to a nitrocellulose membrane and reacted with appropriate primary and secondary antibodies. Protein bands were detected by enhanced chemiluminescence (GE Healthcare). α-Tubulin was used as a loading control.

### Apoptosis assay

The annexin V assay was performed as previously described [33] using an LSR Fortessa flow cytometer (BD Biosciences). To study apoptosis in cell lines, cells were treated with various concentrations of BIX-01294 or other drugs for 48 h, then resuspended in annexin V binding buffer, and incubated with annexin V/FITC (BD Pharmingen) and propidium iodide (PI) or 7-AAD (Beckman Coulter) for 15 min before flow cytometry analysis. To study apoptosis in LSCs from primary AML cells, cells were divided into CD34^+^CD38^−^ fractions by staining with anti-CD34-APC (BD Biosciences), anti-CD38-PE (BD Biosciences), and 7-AAD for 30 min. Data were analyzed using the FACSuite software (BD Biosciences).

### Confocal microscopy

Cells were centrifuged at 800×*g* onto glass slides, and coverslips were mounted with aqueous mounting medium (Dako) containing DAPI (Sigma–Aldrich). Fluorescence signals were analyzed using a Zeiss LSM 700 laser-scanning confocal microscope. LC3 puncta were quantified in cells as described [33]. The average number of LC3 puncta per cell in each treatment group was estimated by manually counting puncta in 20 randomly selected cells.

### Measurement of intracellular generation of ROS

Cells were treated with a given drug alone or in combination with the antioxidant *N*-acetylcysteine [NAC; (*R*)-2-acetamido-3-sulfanylpropanoic acid; Sigma–Aldrich] after preincubation with 10 μmol/L dichlorodihydrofluorescein diacetate (DCFH-DA; Invitrogen) at 37 °C for 30 min. In addition, 1 × 10^5^ cells were stained with 10 μmol/L DCFH-DA at 37 °C for 30 min, then washed, and resuspended in Dulbecco’s phosphate-buffered saline (Gibco Life Technologies). The amount of the dihydrofluorescein formed was measured by flow cytometry.

### Small interfering RNA (siRNA) transfection

siRNAs against PERK, G9a, and NRF2 were purchased from Qiagen. Leukemia cells (2 × 10^6^) were directly transfected with siRNA (1 μmol/L) using the V^− 01^ program on an Amaxa nucleofector device (Lonza Cologne GmbH), according to the manufacturer’s instructions. After electroporation, the cells were resuspended in a complete medium and incubated at 37 °C in a humidified atmosphere containing 5% CO_2_. Control cells were transfected with a scrambled siRNA.

### Transfection of green fluorescent protein (GFP)-tagged LC3

Mammalian GFP-LC3 expression plasmids were described previously [33]. Leukemia cells (2 × 10^6^) were directly transfected with GFP-LC3 cDNA (5 mg), as described above for siRNA. Immediately after electroporation, the cells were resuspended in a complete medium and incubated at 37 °C in a humidified atmosphere containing 5% CO_2_ for 24 h. Cells expressing the GFP-tagged LC3 were used to evaluate autophagy induction. GFP-LC3 dots in each cell were counted in at least three separate visual fields.

### Statistical analysis

Data are expressed as the mean ± standard deviation (SD) of at least three independent experiments. Means of two groups were compared using a two-tailed Student’s *t*-test in GraphPad Prism 4.0 (GraphPad Software, Inc.). *P*-values of less than 0.05 were considered significant.

## Results

### G9a inhibition induced apoptosis in AML cells

**Fig. 1:**
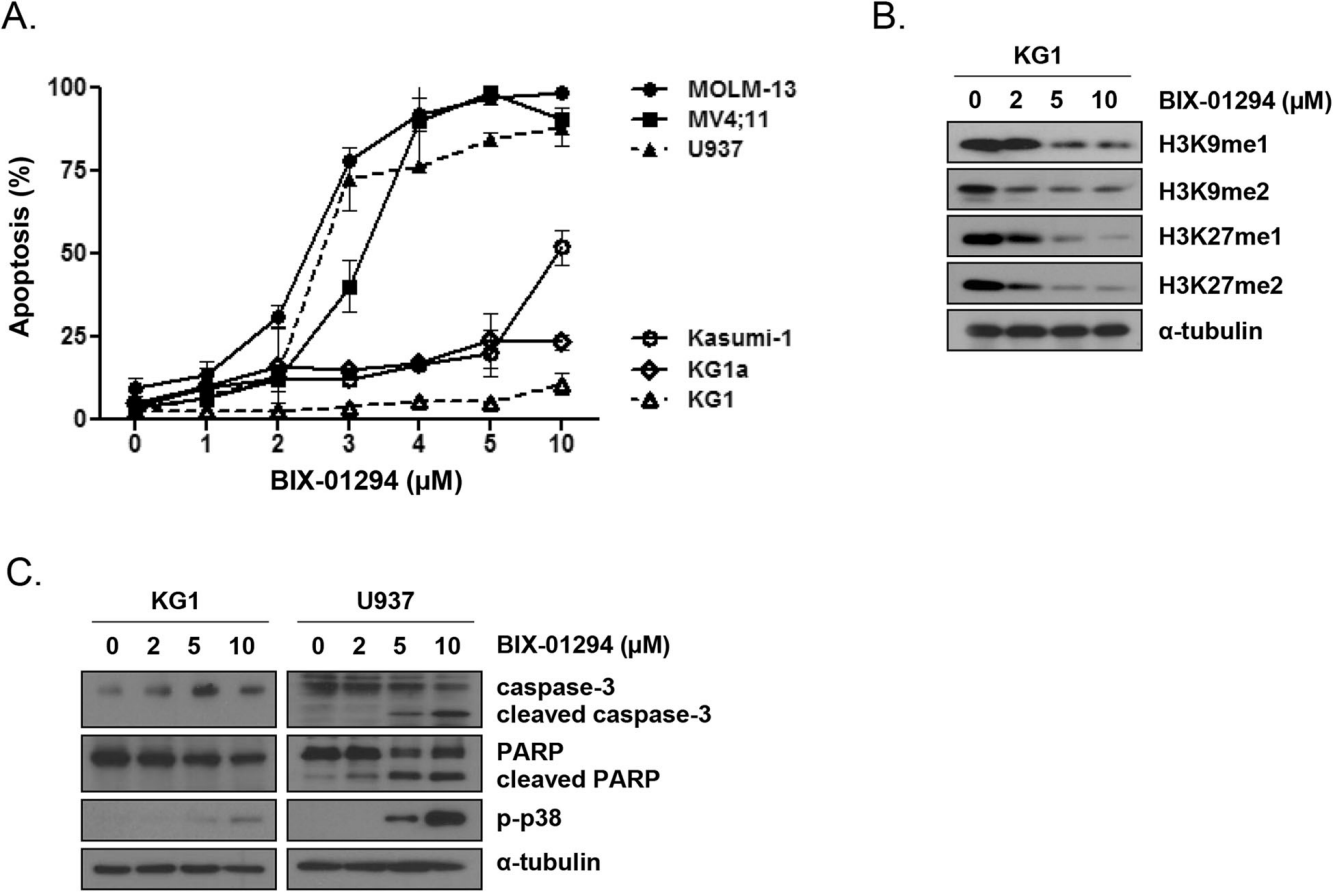
Effects of G9a inhibition in leukemia cells and LSC-like cells. **a**, Flow cytometry analysis, based on annexin V/PI exclusion, of the fraction of apoptotic cells after treatment of AML cells and LSC-like cells with BIX-01294 for 48 h. **b**, H3K9 and H3K27 methylation, analyzed by western blotting, in KG1 cells treated with various concentrations of BIX-01294 for 24 h. **c**, Protein expression, analyzed by western blotting with the indicated antibodies, in KG1 and U937 cells treated with various concentrations of BIX-01294 for 48 h

The apoptotic response to BIX-01294 treatment differed among the AML cell lines evaluated. In MOLM-13, MV4–11, and U937 cells, apoptosis was induced in a concentration-dependent manner. By contrast, in the AML LSC-like cell lines KG1, KG1a, and Kasumi-1, which originated from early myeloid stem cells with more than 70% of CD34^+^ cells [34–36], the extent of BIX-01294-induced apoptosis was lower (Fig. 1a). Among the LSC-like cell lines, KG1 cells were very insensitive to BIX-01294 treatment; therefore, we used these cells as representative BIX-01294-resistant cells in further experiments. BIX-01294 suppressed the levels of mono- and dimethylation of H3K9 (H3K9me1 and H3K9me2) and H3K27 (H3K27me1 and H3K27me2) in a concentration-dependent manner in KG1 cells (Fig. 1b). As cleavage of PARP by activated (cleaved) caspase-3 is a hallmark of apoptosis, we next assessed changes in the levels of these molecules in U937 and KG1 cells after treatment with BIX-01294. As expected, cleaved caspase-3 and PARP were detected after treatment with BIX-01294 in U937 but not in KG1 cells (Fig. 1c). In U937 cells, BIX-01294 treatment markedly induced phosphorylation of p38, which is one of the major mammalian mitogen-activated protein kinases (MAPKs) and is commonly involved in ROS-induced apoptosis [37]. By contrast, p-p38 induction was minimal in KG1 cells (Fig. 1c).

### G9a inhibition induced prosurvival PERK signaling in LSC-like cell lines

**Fig. 2:**
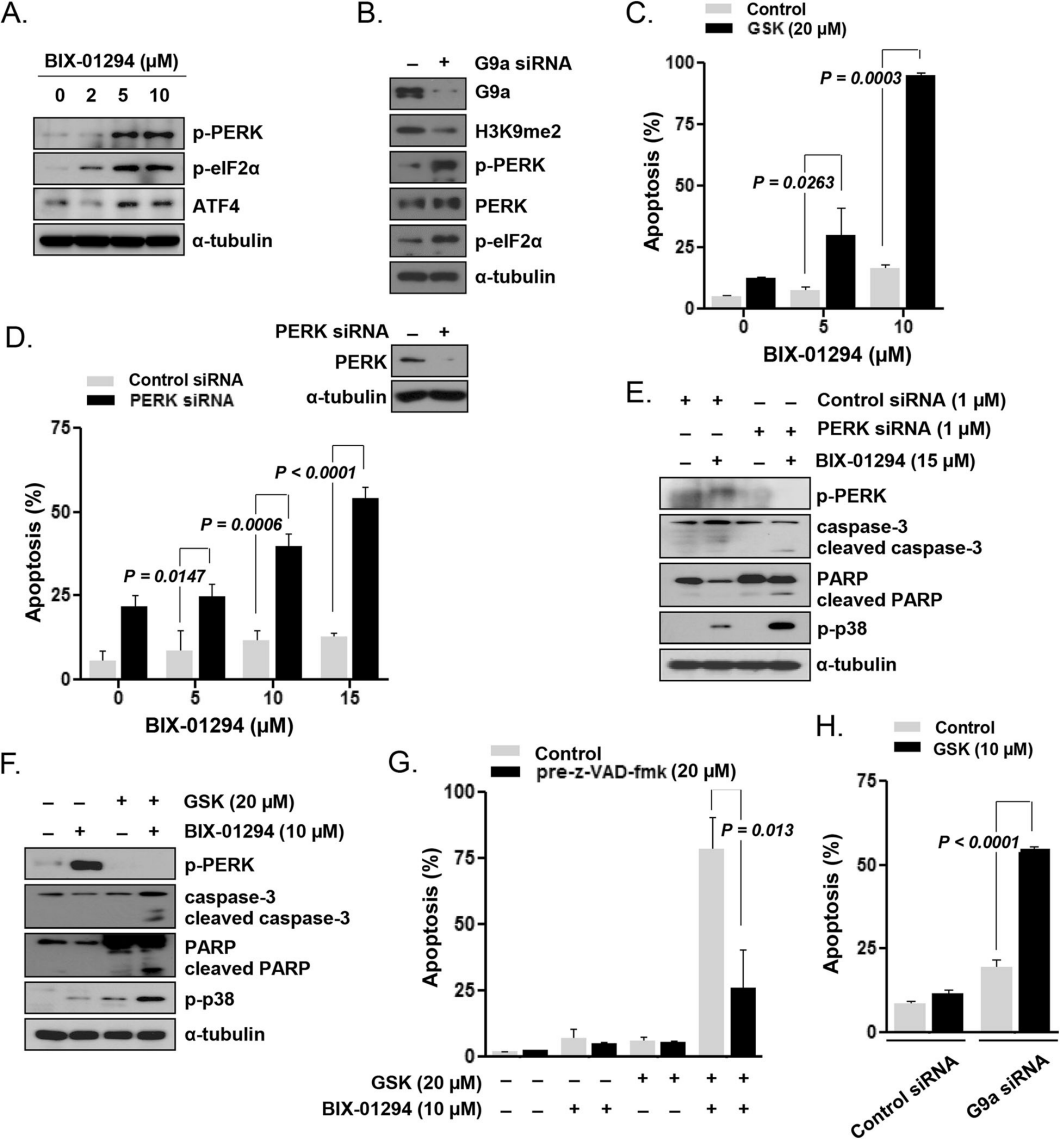
Induction of prosurvival PERK signaling by G9a inhibition in KG1 LSC-like cells. **a**, Expression of PERK pathway proteins, analyzed by western blotting, in cells treated with various concentrations of BIX-01294 for 24 h. **b**, Protein expression, analyzed by western blotting with the indicated antibodies, in cells transfected with G9a-targeting or control siRNA. **c**, Flow cytometric analysis of the apoptotic fraction of cells treated with various concentrations of BIX-01294 in the absence or presence of the PERK inhibitor GSK2606414 (20 μmol/L) for 48 h. **d**, Apoptosis levels, determined by flow cytometry, in cells transfected with PERK-targeting or control siRNA and treated with various concentrations of BIX-01294 for 48 h. **e**, Protein expression, analyzed by western blotting with the indicated antibodies, in PERK/control siRNA-transfected cells after treatment with 15 μmol/L BIX-01294 for 48 h. **f**, Protein expression, analyzed by western blotting with the indicated antibodies, in cells treated with BIX-01294 (10 μmol/L) in the absence or presence of GSK2606414 (20 μmol/L) for 48 h. **g**, Apoptosis levels, determined by flow cytometry, in cells pretreated or not with Z-VAD-FMK (20 mmol/L) for 1 h and then treated with BIX-01294 (10 μmol/L) and GSK2606414 (20 μmol/L) for 48 h. **h**, Apoptosis levels, determined by flow cytometry, in cells transfected with G9a-targeting or control siRNA and incubated with GSK2606414 (10 μmol/L) for 48 h

Next, we examined whether G9a inhibition activates the PERK pathway in KG1 cells. BIX-01294 treatment activated the PERK/eIF2α phosphorylation pathway, with increased ATF4 expression, in KG1 cells (Fig. 2a). Upon siRNA-mediated G9a knockdown, the level of H3K9me2 effectively decreased, and PERK/eIF2α pathway phosphorylation increased (Fig. 2b). To evaluate whether PERK activation contributes to survival of KG1 cells, we examined the effect of PERK inhibition by the pharmacological inhibitor GSK2606414 or via RNA interference. We selected the most effective cytotoxic concentration of GSK2606414 and BIX-01294 to perform a further experiment (Additional file 1: Figure S1). When KG1 cells were cotreated with 20 μmol/L GSK2606414 and 10 μmol/L BIX-01294, apoptosis was synergistically enhanced (*P* = 0.0003; Fig. 2c). The effects of the PERK inhibitor treatment were verified by PERK silencing using siRNA to confirm that these effects were not due to the inhibition of any other kinase with the high concentration of the PERK inhibitor. In KG1 cells transfected with the PERK siRNA, but not in non-transfected control cells, BIX-01294 treatment strongly induced apoptosis in a concentration-dependent manner (Fig. 2d). These results suggested that the prosurvival PERK pathway was activated in AML LSCs upon inhibition of the G9a activity. Similar to our observations in BIX-01294-sensitive U937 cells (Fig. 1c), in KG1 cells, in which PERK was inhibited using siRNA (Fig. 2e) or GSK2606414 (Fig. 2f), BIX-01294 effectively induced p38 phosphorylation and enhanced the levels of cleaved caspase-3 and PARP. Incubation with a pan-caspase inhibitor (Z-VAD-FMK, 20 μmol/L) significantly blocked the apoptosis induction by GSK2606414 plus BIX-01294 in KG1 cells (Fig. 2g). Apoptosis was strongly induced upon treatment with 10 μmol/L GSK2606414 in G9a-knockdown cells but not in non-transfected control KG1 cells (*P* < 0.0001; Fig. 2h). Next, we analyzed the effect of PERK pathway inhibition on BIX-01294-induced apoptosis in KG1a cells, another LSC-like cell line. BIX-01294 and GSK2606414 synergistically enhanced the apoptosis in KG1a cells (Additional file 1: Figure S2A; *P* = 0.0092). siRNA-mediated PERK knockdown also significantly enhanced the BIX-01294-induced apoptosis in KG1a cells (Additional file 1: Figure S2B; *P* = 0.0021).

### PERK inhibition sensitized primary AML LSCs but not HSCs to BIX-01294

**Fig. 3:**
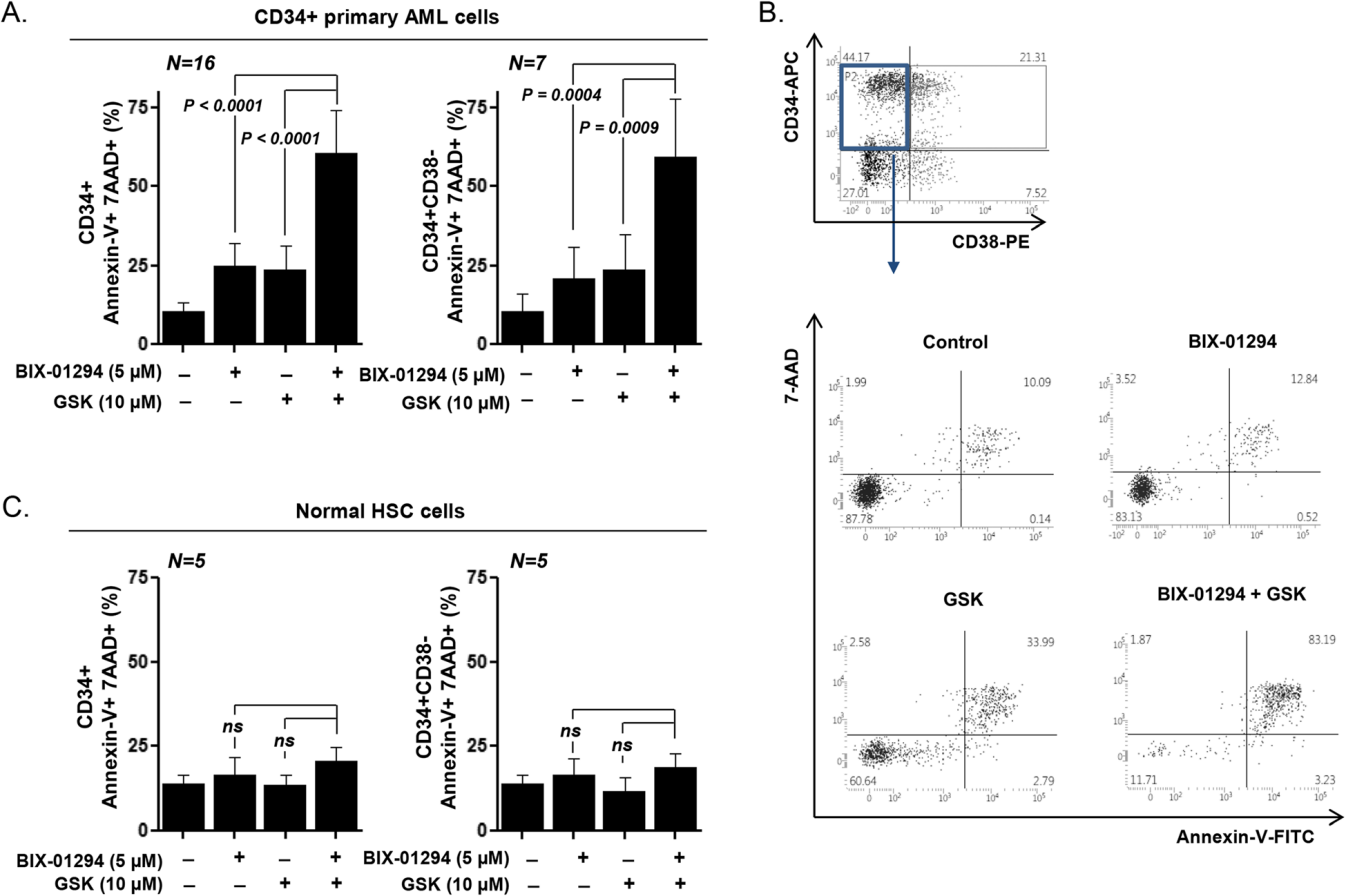
Sensitization of AML LSCs, but not HSCs, to BIX-01294-induced apoptosis via PERK inhibition. **a**, Flow cytometric analysis, based on annexin-V/7-AAD exclusion, of apoptotic fractions after treatment of CD34^+^-enriched primary AML cells from 16 cases with BIX-01294 (5 μmol/L) in the absence or presence of GSK2606414 (10 μmol/L) for 48 h. Left panel: annexin-V^+^ 7-AAD^+^ CD34^+^ cells; right panel: CD34^+^CD38^−^ primary LSCs. **b**, Flow cytometric analysis, based on annexin-V/7-AAD exclusion, of apoptotic fractions after treatment of normal HSCs from five donors with BIX-01294 (5 μmol/L) in the absence or presence of GSK2606414 (10 μmol/L) for 48 h. Left panel: annexin-V^+^ 7-AAD^+^ CD34^+^ cells; right panel: CD34^+^CD38^−^ primary HSCs. **c**, Representative scatter plot of CD34^+^-enriched primary AML cells

Next, we assessed the effect of cotreatment with BIX-01294 and GSK2606414 in CD34^+^-enriched primary AML LSCs and normal HSCs (Fig. 3 and Additional file 1: Table S1). GSK2606414 enhanced the sensitivity of CD34^+^ primary AML cells and significantly enhanced the BIX-01294-induced cell apoptosis (24.79 ± 1.77% for BIX-01294 alone and 60.38 ± 3.39% for BIX-01294 plus GSK2606414; *P* < 0.0001; Fig. 3a, left panel). Moreover, the apoptotic cell fraction (annexin V^+^ 7-AAD^+^) of CD34^+^CD38^−^ primary AML LSCs remarkably increased upon cotreatment with GSK2606414 and BIX-01294 [20.74 ± 3.88% for BIX-01294 alone and 59.36 ± 6.92% for BIX-01294 plus GSK2606414; *P* = 0.0004; Fig. 3a (right panel) and 3B]. By contrast, no significant proapoptotic effect of cotreatment with BIX-01294 and GSK2606414 was observed in HSCs (Fig. 3c, left panel). The effect on normal hematopoietic cells, with a CD34^+^CD38^−^ immunophenotype, was not significant either (Fig. 3c, right panel), suggesting that the PERK-mediated resistance mechanism may act specifically in CD34^+^CD38^−^ LSCs.

### PERK signaling attenuated BIX-01294-induced ROS generation and apoptosis in LSC-like cells

**Fig. 4:**
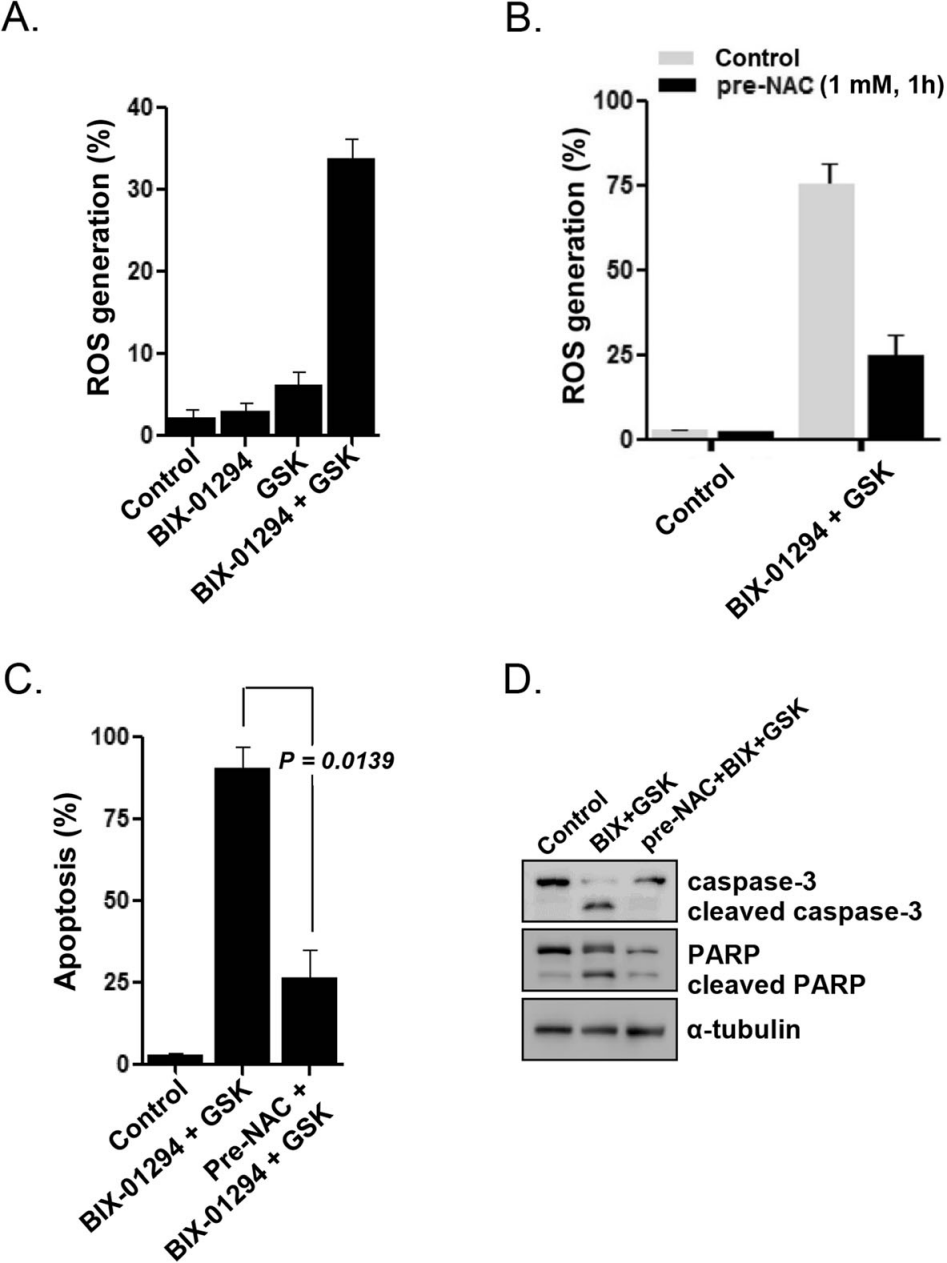
Effects of PERK inhibition on BIX-01294-induced ROS generation and ROS-induced apoptosis of KG1 LSC-like cells. **a**, Intracellular ROS generation, measured by flow cytometry, in cells treated with BIX-01294 (10 μmol/L) in the absence or presence of GSK2606414 (20 μmol/L) for 16 h. **b**, ROS generation after cotreatment of cells with BIX-01294 (10 μmol/L) and GSK260641 (20 μmol/L) with/without preincubation with NAC (1 mmol/L) for 1 h. **c**, Apoptosis levels, determined by flow cytometry, after cotreatment of cells with BIX-01294 (10 μmol/L) and GSK2606414 (20 μmol/L) for 48 h, with/without preincubation with NAC (1 mmol/L) for 1 h. **d**, Protein expression, analyzed by western blotting with the indicated antibodies, after cotreatment of cells with BIX-01294 (10 μmol/L) and GSK2606414 (20 μmol/L) for 48 h, with/without preincubation with NAC (1 mmol/L) for 1 h

Dichlorodihydrofluorescein diacetate (DCFH-DA), which is oxidized by cellular ROS, fluorescence measurement revealed that BIX-01294 treatment alone did not induce ROS generation in KG1 cells (Fig. 4a). However, ROS accumulation was observed after cotreatment with 10 μmol/L BIX-01294 and 20 μmol/L GSK2606414 (Fig. 4a). The antioxidant N-acetylcysteine (NAC) was used to investigate whether the increase in ROS generation upon cotreatment was primarily caused cell death. Pretreatment of KG1 cells with 1 mmol/L NAC significantly suppressed the ROS accumulation (Fig. 4b) and apoptosis (Fig. 4c), which were induced by cotreatment with BIX-01294 and GSK2606414. Cleavage of caspase-3 and PARP was not detected when KG1 cells were treated with BIX-01294 and GSK2606414 after NAC pretreatment (Fig. 4d). These results indicated that PERK inhibition enhanced LSC sensitivity to BIX-01294-induced ROS generation and caspase-dependent apoptosis.

### G9a inhibition-induced PERK signaling conferred resistance to LSCs via NRF2/HO-1 activation

**Fig. 5:**
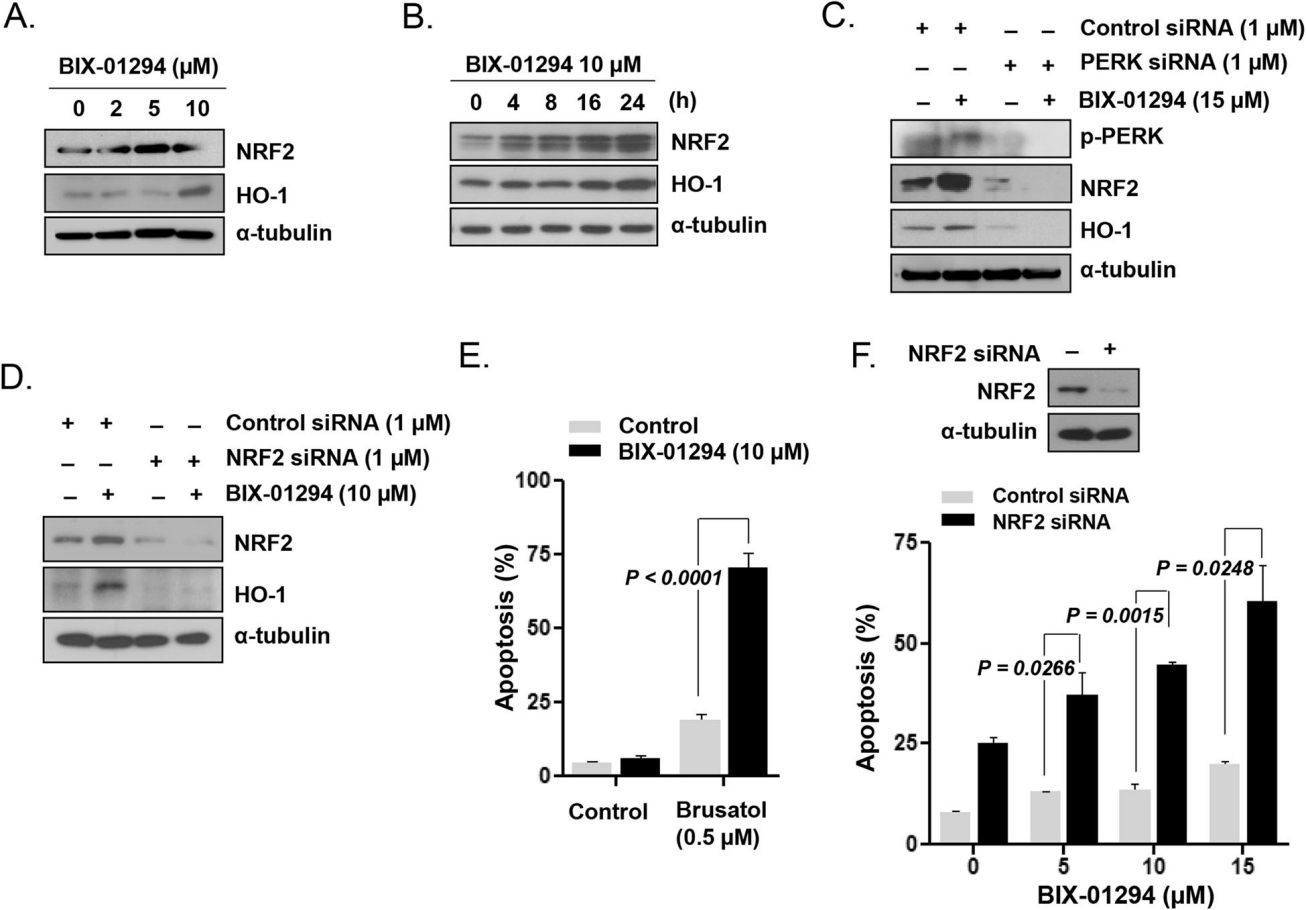
Prosurvival PERK/NRF2/HO-1 activation in KG1 LSC-like cells upon G9a inhibition. **a**, NRF2 and HO-1 expression, analyzed by western blotting, after cell treatment with various concentrations of BIX-01294 for 24 h. **b**, NRF2 and HO-1 expression, analyzed by western blotting, after cell treatment with BIX-01294 (10 μmol/L) for different periods. **c**, Protein expression, analyzed by western blotting with the indicated antibodies, in PERK siRNA-transfected and non-transfected cells after treatment with BIX-01294 (15 μmol/L) for 48 h. **d**, Protein expression, analyzed by western blotting with the indicated antibodies, in NRF2 siRNA-transfected and non-transfected cells after treatment with BIX-01294 (10 μmol/L) for 48 h. **e**, Apoptotic fractions, measured using flow cytometry, after cell treatment with BIX-01294 (10 μmol/L) in the absence or presence of the NRF2 inhibitor brusatol (0.5 μmol/L) for 48 h. **f**, Apoptosis levels, determined by flow cytometry, after treatment of NRF2 siRNA- or control siRNA-transfected cells with different concentrations of BIX-01294 for 48 h

We found that BIX-01294 upregulated NRF2, which is a direct substrate of the PERK kinase, resulting in increased HO-1 expression in KG1 cells (Fig. 5a and b). siRNA-mediated PERK knockdown effectively abolished the BIX-01294-mediated upregulation of NRF2 and HO-1 in KG1 cells (Fig. 5c). HO-1 was downregulated in NRF2-silenced KG1 cells after BIX-01294 treatment (Fig. 5d), indicating that HO-1 is regulated by the PERK/NRF2 pathway. Treatment with brusatol, a specific inhibitor of NRF2, led to a significant increase in the extent of BIX-01294-induced apoptosis in a concentration-dependent manner in KG1 cells (*P* < 0.0001; Fig. 5e and Additional file 1: Figure S3). Apoptosis was significantly enhanced upon treatment of NRF2-knockdown cells but not control siRNA-transfected or non-transfected KG1 cells with 10 μmol/L BIX-01294 (*P* = 0.0015; Fig. 5f).

### G9a inhibition induced PERK pathway-independent prosurvival autophagy in LSCs

**Fig. 6:**
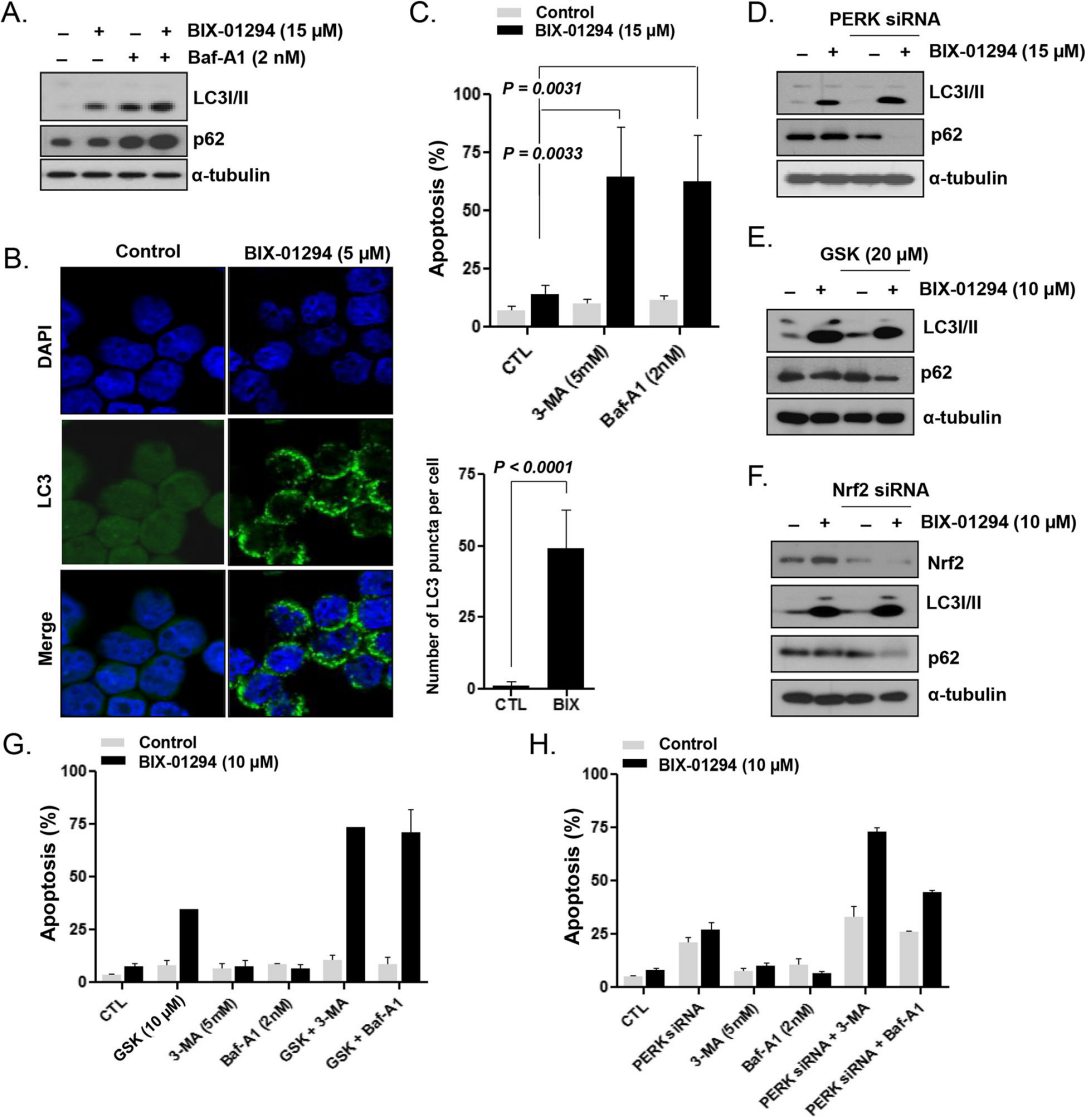
PERK pathway-independent induction of prosurvival autophagy in KG1 LSC-like cells upon G9a inhibition. **a**, LC3-I/II and p62 expression, analyzed by western blotting, after cell treatment with BIX-01294 (15 μmol/L) in the absence or presence of bafilomycin A1 (BafA1) for 24 h. **b**, Confocal microscopy images of GFP-LC3-transfected cells after treatment with BIX-01294 (5 μmol/L). **c**, Apoptotic fractions, measured using flow cytometry, after cell treatment with BIX-01294 (15 μmol/L) in the absence or presence of the autophagy inhibitor 3-MA (5 mmol/L) or BafA1 (2 nmol/L) for 48 h. **d**, Protein expression, analyzed by western blotting with the indicated antibodies, after treatment of PERK siRNA-transfected and non-transfected cells with BIX-01294 (15 μmol/L) for 48 h. **e**, Protein expression, analyzed by western blotting with the indicated antibodies, after cell treatment with BIX-01294 (10 μmol/L) in the absence or presence of GSK2606414 (20 μmol/L) for 48 h. **f**, Protein expression, analyzed by western blotting with the indicated antibodies, after treatment of NRF2 siRNA-transfected and non-transfected cells with BIX-01294 (10 μmol/L) for 48 h. **g**, Levels of apoptosis, evaluated by flow cytometry, after cell treatment for 48 h with BIX-01294 (10 μmol/L) in the absence or presence of the PERK inhibitor GSK2606414 (10 μmol/L) or the autophagy inhibitor 3-MA (5 mmol/L), or BafA1 (2 nmol/L). **h,** Levels of apoptosis, evaluated by flow cytometry, after treatment of PERK siRNA- or control siRNA-transfected cells with BIX-01294 (10 μmol/L) in the absence or presence of 3-MA (5 mmol/L) or BafA1 (2 nmol/L)

Next, we evaluated the association between prosurvival PERK activation and autophagy in KG1 cells. The LC3-II level increased in KG1 cells after BIX-01294 treatment (Fig. 6a). After the addition of bafilomycin A1, an inhibitor of autophagosome degradation, the BIX-01294-induced LC3-II formation was further augmented and p62 was accumulated, compared with the respective levels observed with BIX-01294 treatment alone (Fig. 6a), indicating that BIX-01294 promoted the autophagic flux in KG1 cells. Microscopic images revealed that BIX-01294 treatment induced a more than 10-fold increase in the number of GFP-LC3 puncta in KG1 cells (*P* < 0.0001; Fig. 6b). The addition of 3-MA or bafilomycin A1 during treatment with 15 μmol/L BIX-01294 significantly increased the level of apoptosis in KG1 cells (3-MA: *P* = 0.0033; bafilomycin A1: *P* = 0.0031; Fig. 6c) compared with that induced by BIX-01294 treatment alone, indicating the occurrence of prosurvival autophagy in LSCs. After PERK inhibition using PERK siRNA (Fig. 6d) or GSK2606414 (Fig. 6e), BIX-01294 treatment still increased the level of LC3-II and induced p62 degradation. Upon the addition of bafilomycin A1, autophagic flux was demonstrated after PERK silencing and BIX-01294 treatment, based on the augmented level of LC3-II (Additional file 1: Figure S4). BIX-01294 treatment after siRNA-mediated NRF2 knockdown increased the level of LC3-II and resulted in p62 degradation (Fig. 6f). These results indicated that BIX-01294 induced prosurvival autophagy even after PERK inhibition, suggesting that autophagy induction occurred independently of the PERK pathway. When we combined treatment with 3-MA or bafilomycin A1 with PERK inhibition using GSK2606414 (Fig. 6g) or PERK siRNA (Fig. 6h), 10 μmol/L BIX-01294 markedly increased apoptosis of KG1 cells, compared with the levels observed with combination treatments with 10 μmol/L BIX-01294 and either 3-MA, bafilomycin A1, GSK2606414, or PERK siRNA.

## Discussion

LSCs are responsible for the initiation, progression, and therapeutic resistance of leukemia. G9a has emerged as a promising target for cancer stem cells [18]. However, the effect of G9a inhibition on LSCs, which develop resistance to therapy via several cell survival mechanisms, has not been explored. To our knowledge, this is the first study demonstrating that resistance to G9a inhibition occurs through PERK/NRF2 signaling and autophagy activation in AML LSC-like cells. G9a inhibition by either BIX-01294 or siRNA triggered activation of the prosurvival PERK/NRF2 pathway in LSC-like cells. Meanwhile, inhibition of PERK/NRF2 signaling remarkably enhanced the G9a-induced apoptosis of LSC-like cell lines and primary CD34^+^CD38^−^ blasts from patients with AML. Autophagy inhibition also significantly increased the level of apoptosis, which was induced by G9a inhibition in LSC-like cells.

This study revealed that LSC-like cells developed resistance to G9a inhibition, while apoptosis was effectively induced in leukemia cell lines. Although a G9a inhibitor effectively inhibited G9a in a concentration-dependent manner, as reflected by the reduced H3K9me2 level in KG1 cells, the inhibitor showed a low efficacy in inducing apoptosis. p38 was strongly phosphorylated in U937 cells, in which apoptosis was significantly induced upon G9a inhibition. By contrast, p38 phosphorylation was very weak in KG1 cells. Accumulated ROS activate protein kinase B (also known as Akt) and apoptosis signal-regulating kinase 1 (ASK1), a serine/threonine kinase that was initially discovered as a MAPK kinase kinase in the p38 MAPK signaling cascade [38]. ASK1 activation results in downstream activation of the p38 MAPK and, eventually, in apoptosis [39, 40]. In this study, suppression of the PERK activation by G9a inhibition in KG1 cells resulted in increased levels of p-p38, which, in turn, enhanced apoptosis, suggesting ROS involvement in this process. We also observed that cotreatment with G9a and PERK inhibitors resulted in increased ROS generation, in contrast to the treatment with the G9a inhibitor alone. After pretreatment with 1 mmol/L NAC, simultaneous inhibition of G9a and PERK did not induce apoptosis of KG1 cells. This finding demonstrated that the activation of the PERK pathway was a critical defense regulatory mechanism against ROS accumulation, which induces apoptosis via p38 MAPK signaling.

To induce death by cotreating LSC-like cells, which survived despite G9a inhibition, with the PERK inhibitor GSK2606414, a high (e.g., 20 μmol/L) concentration of the inhibitor was required in this study. Because GSK2606414, as all kinase inhibitors, is directed to the ATP-binding site of PERK, it shows off-target kinase inhibitor effects. To confirm that the effects of GSK2606414 at the high concentration were not due to the inhibition of any other kinase nor to off-target effects, PERK was also silenced with siRNA. Despite the subnanomolar IC_50_ of GSK2606414, higher concentrations of GSK2606414 were needed to completely block PERK autophosphorylation under conditions of extreme ER stress [41]. Presumably, PERK activation was extremely induced by ROS when G9a was inhibited in LSC-like cells, and therefore, a higher concentration of the PERK inhibitor was required to suppress the PERK pathway.

Whereas PERK signaling is largely mediated through the eIF2α/ATF4 pathway, the transcription factor NRF2 is also a direct substrate of the PERK kinase [42], with ATF4 interacting with NRF2 to regulate HO-1 expression. During ER stress, PERK phosphorylates NRF2, leading to its nuclear translocation, and contributes to cellular redox homeostasis by upregulating the antioxidant HO-1 [42, 43]. Consistently, we found that G9a inhibition induced PERK phosphorylation, leading to eIF2α/ATF4 and NRF2 activation in LSC-like cells. In addition, our data showed that the PERK/NRF2 pathway was activated, leading to the upregulation of HO-1 expression, thus protecting LSC-like cells against oxidative stress.

Cotreatment with G9a and PERK inhibitors synergistically enhanced apoptosis of LSCs, while sparing HSCs, which respond to oxidative stress differently than LSCs do [44]. Low levels of ROS are required to maintain quiescence and stemness of HSCs, whereas increased ROS levels trigger myeloid progenitor differentiation of these cells [45]. In contrast, LSCs show peculiar metabolic properties, being more dependent on oxidative respiration than on glycolysis; moreover, LSCs are more sensitive to oxidative stress than HSCs are [44]. The different responses to G9a inhibition-induced ROS may be explained by these characteristics of LSCs and HSCs. These results suggest that the ROS accumulation, induced by targeting PERK/NRF2, opens up a possibility of selective LSC eradication. Rushworth et al. have reported that NRF2 knockdown significantly augmented the chemotherapy-induced reduction in colony formation by AML cells [46], which may be explained by our findings.

Anticancer therapies commonly lead to the activation of prosurvival autophagy, allowing cancer cells to overcome cytotoxicity or other treatment-induced stresses [47]. We previously reported that autophagy induction was involved in the resistance of AML cells to cytosine arabinoside [48] and LSCs to a bromodomain and extraterminal domain inhibitor [33], which provided evidence that autophagy could act as a prosurvival mechanism in AML. Consistently, in this study, we observed a protective autophagic response in LSC-like cells after BIX-01294 treatment. The addition of autophagy inhibitors to G9a inhibitor treatment resulted in a significant increase in the level of apoptosis of AML LSC-like cells. Thus, our findings indicate that autophagy induction is one of the critical mechanisms involved in BIX-01294 resistance in AML LSCs. Previous studies have shown that the autophagy pathway is induced in response to G9a inhibitor treatment in breast, bladder, and colon cancers [19, 26, 49]. These studies suggested that autophagy induction by G9a inhibition functioned as a prodeath and not prosurvival signal in cancer cells, while the opposite was observed in LSCs in our study. Cancer cells have heterogeneous properties, depending on the cell type, and thus, either induction or inhibition of autophagy can provide therapeutic benefits [50].

ER stress is considered to activate autophagy via UPR-mediated transcriptional upregulation of components of the autophagic machinery and the modulation of LC3 [51]. Induction of the PERK pathway activates an ER stress-induced autophagic mechanism, which plays an important role in cell survival [52]. Alterations in the expression/activity of key UPR components (i.e., IRE1, PERK, and related transducers) and induction of prosurvival autophagy (in an IRE1- and PERK-dependent manner) have been reported in tumor cells [51]. However, when we investigated the interaction between the PERK pathway and autophagy by evaluating the expression of autophagy-associated molecules after PERK/NRF2 inhibition, we found that G9a inhibition-induced autophagy and PERK/NRF2 were not interrelated in LSCs. Further investigation of the regulatory mechanism of prosurvival autophagy is needed to improve our knowledge of the mechanism of resistance of AML LSCs to G9a inhibition.

## Conclusions

**Fig. 7:**
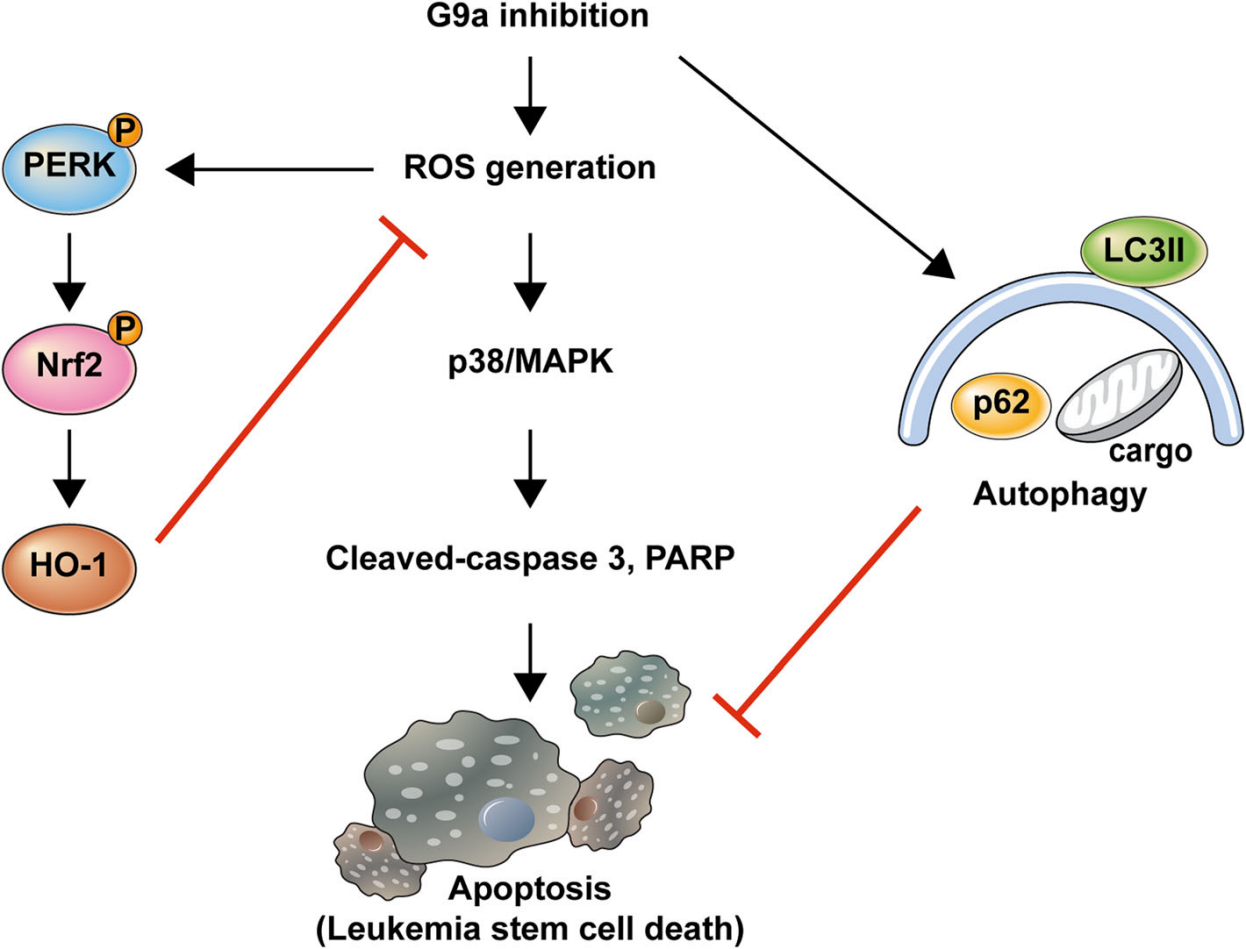
Diagram illustrating potential effects of G9a inhibition in LSCs

This is the first study providing evidence that inhibition of the ROS-activated PERK/NRF2 pathway and of ER stress-independent autophagy augments caspase-dependent apoptosis, which is induced by G9a inhibition in LSCs. Based on these findings, we propose a model to explain the resistance of LSCs to G9a inhibition (Fig. 7). These results suggest that combining PERK/NRF2 and autophagy inhibition with G9a-targeting treatment may be a useful approach for LSC elimination, potentially leading to curative therapies for AML. Differential expression of or mutations in genes associated with PERK/NRF2 activation may serve as prognostic markers in AML. However, future studies are needed to determine how these cellular processes are differentially regulated in LSCs. Additionally, in vivo studies are needed to confirm the effects of the combination of PERK/NRF2 pathway or autophagy inhibition with that of G9a in LSCs. Our findings may provide valuable information on the underlying molecular mechanism of resistance of LSCs to G9a inhibition, along with potential therapeutic targets to overcome AML resistance to G9a inhibitors and to eradicate LSCs.

## Supplementary information

**Additional file 1: Table S1.** Synergistic effects of the G9a and PERK inhibitor on apoptosis of primary acute myeloid LSCs. **Figure S1.** Effects of treatment with the PERK inhibitor GKS2606414 for 48 h in the presence or absence of 10 μM BIX-01294, on apoptosis. **Figure S2.** Effect of PERK inhibition on BIX-01294- induced apoptosis in KG1a cells. (A) KG1a cells were treated with 10 μM BIX-01294 in the presence or absence of the PERK inhibitor GSK260641 at 5 μM. After incubation for 48 h, the apoptotic fraction was measured using flow-cytometric analysis. (B) KG1a cells were transfected with PERK siRNA or scrambled siRNA as described in the Materials and Methods and then treated with 10 μM BIX-01294 for 48 h. **Figure S3.** Effects of treatment for 48 h with the NRF2 inhibitor brusatol in the presence or absence of 10 μM BIX-01294 on apoptosis. **Figure S4.** Effects of PERK inhibition in the absence or presence of 2 nM bafilomycin A1 on autophagy induction.

## Data Availability

The analyzed data sets generated during the study are available from the corresponding author on reasonable request.

## Abbreviations

AML: Acute myeloid leukemia
LSC: Leukemia stem cell
ROS: Reactive oxygen species
HMTase: Histone methyltransferase
H3K9me2: Dimethylation of histone H3 lysine 9
ER: Endoplasmic reticulum
UPR: Unfolded protein response
HSC: Hematopoietic stem cell
3-MA: 3-methyladenine
LC3: Microtubule-associated protein 1 light chain 3
PARP: Poly (ADP-ribose) polymerase
NAC: N-acetylcysteine
siRNA: Small interfering RNA
GFP: Green fluorescent protein
MAPKs: Mitogen-activated protein kinases
ASK1: Apoptosis signal-regulating kinase 1
