# Supplementary material for: PERK/NRF2 and autophagy form a resistance mechanism against G9a inhibition in leukemia stem cells
Source: J Exp Clin Cancer Res. 2020 Apr 15;39:66. doi: 10.1186/s13046-020-01565-3 (PMC7158163; doi:10.1186/s13046-020-01565-3)
Supplement: Supplementary file 1 — Additional file 1: Table S1. Synergistic effects of the G9a and PERK inhibitor on apoptosis of primary acute myeloid LSCs. Figure S1. Effects of treatment with the PERK inhibitor GKS2606414 for 48 h in the presence or absence of 10 μM BIX-01294, on apoptosis. Figure S2. Effect of PERK inhibition on BIX-01294- induced apoptosis in KG1a cells. (A) KG1a cells were treated with 10 μM BIX-01294 in the presence or absence of the PERK inhibitor GSK260641 at 5 μM. After incubation for 48 h, the apoptotic fraction was measured using flow-cytometric analysis. (B) KG1a cells were transfected with PERK siRNA or scrambled siRNA as described in the Materials and Methods and then treated with 10 μM BIX-01294 for 48 h. Figure S3. Effects of treatment for 48 h with the NRF2 inhibitor brusatol in the presence or absence of 10 μM BIX-01294 on apoptosis. Figure S4. Effects of PERK inhibition in the absence or presence of 2 nM bafilomycin A1 on autophagy induction. [file 13046_2020_1565_MOESM1_ESM.pdf]

Table S1. Synergistic effects of the G9a inhibitor and PERK inhibitor on apoptosis of primary acute myeloid LSCs

Patient No.	Sex/ Age (years)	Chromo-some	Mutation		CD34, %	BIX (5 μM, 48 h)		GSK (10 μM, 48 h)		BIX+GSK	
			FLT3-ITD	*NPM1*		Annexin V^+ $7AAD^+$ of $CD34^+$, %	Annexin V^+ $7AAD^+$ of $CD34^+CD38^-$, %	Annexin V^+ $7AAD^+$ of $CD34^+$, %	Annexin V^+ $7AAD^+$ of $CD34^+CD38^-$, %	Annexin V^+ $7AAD^+$ of $CD34^+$, %	Annexin V^+ $7AAD^+$ of $CD34^+CD38^-$, %
YH03	M/48	46,XY[20]	Neg	Neg	73.3	14.7	-	21.7	-	41.0	-
YH04	M/41	46,XY[20]	Neg	Neg	98.4	21.4	-	26.5	-	48.3	-
YH05	M/68	46,XY[20]	Neg	Neg	73.8	26.2	15.1	16.5	22.7	71.2	51.5
YH06	F/74	46,XX[20]	Neg	Neg	81.0	19.5	-	20.7	-	49.0	-
YH08	F/39	46,XX[20]	Pos	Neg	95.1	23.8	-	24.5	-	61.2	-
YH09	M/46	46,XX[15]	Pos	Neg	76.9	24.8	-	31.2	-	63.4	-
YH10	M/39	46,XY[20]	Neg	Neg	74.0	29.2	-	27.3	-	65.4	-
YH11	M/56	46,XY[10]	Neg	Neg	92.1	19.1	11.9	28.3	32.9	70.0	76.2
YH12	F/79	46,XX[22]	Neg	Neg	73.5	33.5	34.0	32.1	42.7	82.3	83.2
YH13	M/61	46,XY[21]	Pos	Neg	94.7	14.9	-	35.6	-	80.6	-
YH14	F/67	46,XX[22]	Neg	NA	65.1	38.6	34.2	21.4	16.1	73.2	68.4
YH15	F/18	46,XX[24]	Neg	Neg	67.3	19.7	-	15.6	-	45.9	-
YH16	F/59	46,XX[23]	Neg	Neg	59.0	19.0	9.3	28.1	23.7	46.2	31.5
YH18	M/70	46,XY[20]	Pos	NA	58.1	29.8	-	8.1	-	44.6	-
YH19	M/24	46,XY[20]	Pos	NA	96.8	34.6	24.4	29.0	17.7	70.8	60.8
YH20	M/75	46,XY[20]	NA	NA	57.3	27.9	16.3	10.5	8.8	53.1	43.9
Donor01	M/25	—	—	—	85.6	15.7	15.2	17.6	16.9	21.7	22.0
Donor02	M/60	—	—	—	68.8	14.7	16.7	12.3	7.4	17.0	13.9
Donor03	M/64	—	—	—	59.4	22.7	22.3	10.4	8.2	25.3	23.1
Donor04	M/58	—	—	—	60.2	20.9	19.2	15.7	14.4	23.8	19.5
Donor05	M/19	—	—	—	60.5	9.6	9.8	10.9	11.6	14.9	16.2

Neg, negative; Pos, positive; NA, not applicable; BM, bone marrow

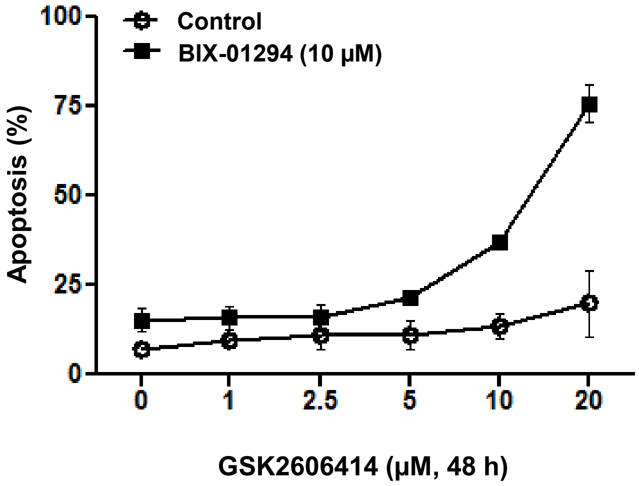

Supplementary Figure S1. KG1 cells were incubated with various concentrations of GSK2606414 for 48 h in the presence or absence of 10 µM BIX-01294, and the fraction of apoptotic cells was analyzed using Annexin-V/PI exclusion and flow-cytometric analysis.

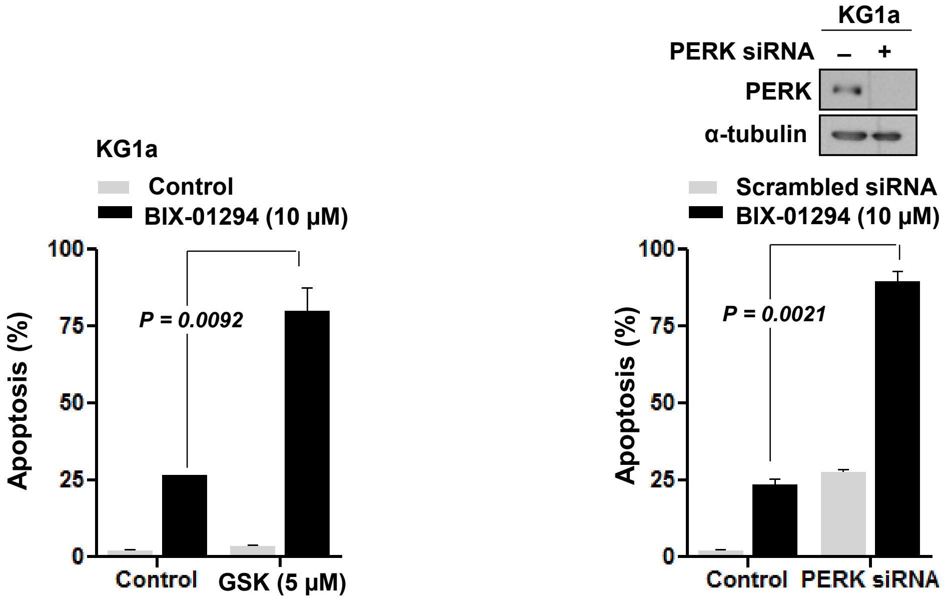

Supplementary Figure S2. (A) KG1a cells were treated with 10 μM BIX-01294 in the presence or absence of the PERK inhibitor GSK260641 at 5 μM. After incubation for 48 h, the apoptotic fraction was measured using Annexin-V/PI-based flow-cytometric analysis. Data are the means ± SDs of three independent experiments. (B) For PERK inhibition experiments, KG1a cells were transfected with PERK siRNA or scrambled siRNA as described in the Materials and Methods. KG1 cells were treated with 10 μM BIX-01294 for 48 h in the absence or presence of PERK siRNA. Scrambled siRNA was used as a control. The levels of apoptosis were determined using flow-cytometric analyses. Data are the means ± SDs of three independent experiments.

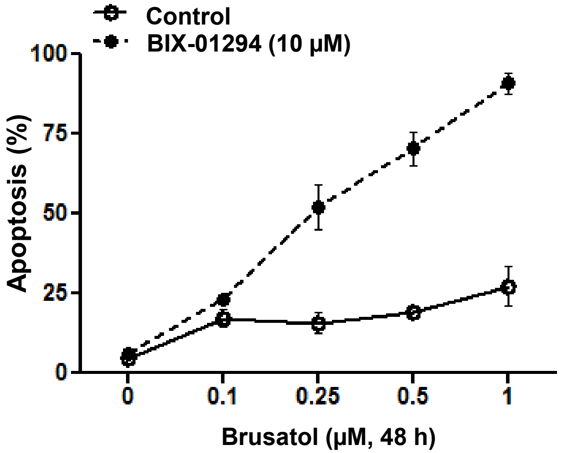

Supplementary Figure S3. KG1 cells were incubated with various concentrations of brusatol for 48 h in the presence or absence of 10 μM BIX-01294, and the fraction of apoptotic cells was analyzed using Annexin-V/PI exclusion and flow-cytometric analysis.

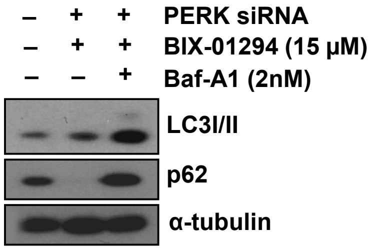

Supplementary Figure S4. KG1 cells were transfected with PERK siRNA or scrambled siRNA and treated with 10 μM BIX-01294 for 24 h in the absence or presence of 2 nM bafilomycin A1. Scrambled siRNA was used as a control. After incubation, cell lysates were subjected to western blotting using the indicated antibodies.
